# Cellular Senescence: The Driving Force of Musculoskeletal Diseases

**DOI:** 10.3390/biomedicines12091948

**Published:** 2024-08-26

**Authors:** Angela Falvino, Beatrice Gasperini, Ida Cariati, Roberto Bonanni, Angela Chiavoghilefu, Elena Gasbarra, Annalisa Botta, Virginia Tancredi, Umberto Tarantino

**Affiliations:** 1 Department of Biomedicine and Prevention, “Tor Vergata” University of Rome, Via Montpellier 1, 00133 Rome, Italy; angelafalvino95@gmail.com (A.F.); beatrice.gasp95@gmail.com (B.G.); roberto.bonanni1288@gmail.com (R.B.); botta@med.uniroma2.it (A.B.); 2 Department of Systems Medicine, “Tor Vergata” University of Rome, Via Montpellier 1, 00133 Rome, Italy; tancredi@uniroma2.it; 3 Department of Orthopaedics and Traumatology, “Policlinico Tor Vergata” Foundation, Viale Oxford 81, 00133 Rome, Italy; angela.chiavo@hotmail.com (A.C.); gasbarra@med.uniroma2.it (E.G.); umberto.tarantino@uniroma2.it (U.T.); 4 Centre of Space Bio-Medicine, “Tor Vergata” University of Rome, Via Montpellier 1, 00133 Rome, Italy; 5 Department of Clinical Sciences and Translational Medicine, “Tor Vergata” University of Rome, Via Montpellier 1, 00133 Rome, Italy

**Keywords:** cellular senescence, musculoskeletal diseases, osteoporosis, sarcopenia, osteoarthritis, SASP, physiology, exercise, nutrition, prevention

## Abstract

The aging of the world population is closely associated with an increased prevalence of musculoskeletal disorders, such as osteoporosis, sarcopenia, and osteoarthritis, due to common genetic, endocrine, and mechanical risk factors. These conditions are characterized by degeneration of bone, muscle, and cartilage tissue, resulting in an increased risk of fractures and reduced mobility. Importantly, a crucial role in the pathophysiology of these diseases has been proposed for cellular senescence, a state of irreversible cell cycle arrest induced by factors such as DNA damage, telomere shortening, and mitochondrial dysfunction. In addition, senescent cells secrete pro-inflammatory molecules, called senescence-associated secretory phenotype (SASP), which can alter tissue homeostasis and promote disease progression. Undoubtedly, targeting senescent cells and their secretory profiles could promote the development of integrated strategies, including regular exercise and a balanced diet or the use of senolytics and senomorphs, to improve the quality of life of the aging population. Therefore, our review aimed to highlight the role of cellular senescence in age-related musculoskeletal diseases, summarizing the main underlying mechanisms and potential anti-senescence strategies for the treatment of osteoporosis, sarcopenia, and osteoarthritis.

## 1. Introduction

The world’s population is experiencing an unprecedented aging trend, with profound implications for economies worldwide [1,2]. This demographic shift coincides with a decline in healthy lifestyles and contributes to the onset of osteoporosis, sarcopenia, and osteoarthritis, three common musculoskeletal disorders that often coexist in the elderly population sharing genetic, endocrine, and mechanical risk factors [3,4,5].

The loss of bone and muscle mass and the progressive degradation of cartilage are the macroscopic effects of the complex pathological processes underlying these diseases, in association with an increased susceptibility to fractures and an elevated risk of falls [6,7]. From a microscopic point of view, affected tissues are characterized by numerous cellular and molecular alterations that induce a state of replicative senescence, irreversibly compromising the quality of the musculoskeletal system [8]. Not surprisingly, cellular senescence has recently emerged as a critical element in the pathophysiology of osteoporosis, sarcopenia, and osteoarthritis [9,10], highlighting the need for further studies to understand the intricate relationship between cellular senescence and musculoskeletal functions, as well as to develop effective strategies to mitigate and manage these debilitating conditions. Cellular senescence, characterized by an irreversible arrest of the cell cycle, is known to be triggered by various factors, including DNA damage response, telomere shortening, mitochondrial dysfunction, and alterations in epigenetic regulation, resulting in morphological, functional, and metabolic changes, as well as resistance to apoptosis [11]. Interestingly, senescent cells exhibit an altered secretome, called senescence-associated secretory phenotype (SASP), which can perform autocrine, paracrine, or endocrine functions with a dual influence on normal biological processes [12]. Positive aspects include wound healing, embryogenesis, and tumor suppression through cell cycle arrest. Indeed, in a young individual, senescent cells release SASPs after an injury, stimulating the immune system to eliminate them. This mechanism simultaneously triggers tissue regeneration by enhancing the stemness of neighboring cells and promoting their proliferation and differentiation [13]. On the other hand, with advancing age, senescent cells accumulate, stimulating the persistent production of SASPs and triggering inflammatory processes [14]. Moreover, chronic exposure to SASPs can prevent cell proliferation and induce senescence in neighboring cells, sustaining a prolonged state of dedifferentiation that hinders tissue regeneration after injury. Thus, the gradual accumulation of senescent cells and related SASPs during the aging process contributes to impaired tissue function and the onset of various diseases, including musculoskeletal disorders [11].

Interestingly, cellular senescence has been suggested to be among those responsible for the decline in regenerative function observed in muscle and bone stem cells with advancing age [15] because of the up-regulation of certain proteins, including p16, p21, and p27, responsible for altered tissue metabolism [5]. Particularly, senescent bone cells are known to release SASPs that promote osteoclast activity, inducing bone resorption and accelerating bone mass loss [16]. In agreement, studies in mouse models have shown that the elimination of senescent cells improves bone mineral density and bone microarchitecture, counteracting the onset of osteoporosis [17]. Furthermore, in skeletal muscle, the senescence of satellite cells, which are essential for muscle regeneration, significantly reduces tissue repair capacity [18]. In this regard, experiments in mouse models have shown that the elimination of senescent cells improves muscle function and increases muscle mass, suggesting senolytics as potential strategies in the treatment of sarcopenia [19]. Finally, the accumulation of senescent cells in joints has been suggested to contribute to cartilage degradation and synovial inflammation, exacerbating the joint deterioration that characterizes osteoarthritis [20].

Overall, senescent cells appear to play a key role in bone and muscle function, highlighting the need for further investigation to explore the underlying mechanisms. Therefore, our review aims to highlight the emerging role of cellular senescence in age-related musculoskeletal diseases, shedding light on potential therapeutic strategies for the intervention and management of these conditions.

## 2. Cellular Senescence in Musculoskeletal Diseases

Cellular senescence plays a key role in musculoskeletal diseases, contributing to conditions like osteoporosis, sarcopenia, and osteoarthritis. In osteoporosis, senescent osteocytes disrupt bone remodeling, reducing bone density and strength [21]. In sarcopenia, senescent muscle cells and their secretory phenotype impair muscle function and regeneration [22]. Similarly, in osteoarthritis, senescent chondrocytes and synovial cells drive inflammation and cartilage degeneration [23]. Undoubtedly, senescence is a critical factor in musculoskeletal aging and disease progression. Therefore, targeting senescent cells and their signaling pathways represents a promising avenue for future therapeutic strategies in the management of these debilitating conditions. The following sections delve into the specific impacts of cellular senescence on bone, muscle, and cartilage health, summarizing the main underlying molecular mechanisms.

### 2.1. Bone Senescence in Osteoporosis

The maintenance of the body’s structural integrity and mechanical strength is ensured by bone tissue that undergoes constant remodeling, characterized by the resorption of damaged or aging bone by osteoclasts followed by the deposition of new bone matrix by osteoblasts [24]. However, with advancing age, a disruption of this balance occurs in the bone microenvironment, causing a decrease in mineral density, strength, and bone mass and predisposing to the onset of osteoporosis [25].

In recent years, a role for cellular senescence has been suggested in the alteration of bone turnover, as the secretory factors released by senescent cells would seem to hinder bone formation and increase bone resorption, promoting skeletal fragility [26]. Furthermore, cellular senescence appears to have a significant impact on fracture healing, which is often delayed and accompanied by a reduction in mesenchymal progenitor cells (MPCs) in the elderly [27]. In this context, recent studies demonstrated a rapid increase of senescent cells in the fracture callus of aged mice compared to non-fracture sites of the same mice and the fracture callus of young mice [28]. This accumulation was associated with inhibition of growth and proliferation of callus-derived MPCs (CaMPCs) and high transforming growth factor beta (TGF-β) levels, a major component of SASP. Noteworthy, an accumulation of senescent cells was also observed in young mice with acute injury, suggesting that the combination of acute injury and natural aging may strongly activate cellular senescence [28].

In addition, age-related bone loss would appear to be largely due to the dysfunction of osteocytes, which, with aging, become senescent cells that are insensitive to mechanical stimuli, resulting in impaired bone remodeling [29]. In this context, it has been demonstrated that various cell types become senescent in the bone microenvironment with normal aging, although senescent osteocytes and senescent myeloid cells are the main sources of SASP [30]. Interestingly, the expression levels of p16, p21, and p53, known effectors of chronic senescence, are increased in osteocytes isolated from 6-month-old young mice compared to 24-month-old aged mice, in association with other key components of SASPs, including interleukin-6 (IL-6), interleukin-8 (IL-8), monocyte chemoattractant protein-1 (MCP-1), tumor necrosis factor α (TNF-α), and various matrix metalloproteinases (MMPs) [30]. A close association between reduced osteocyte numbers and faster bone aging has also been suggested by Ding and colleagues. Indeed, partial ablation of the α-subunit of diphtheria toxin in dentin matrix protein 1 (DMP1)-positive osteocytes in a mouse model of conditional osteocyte deletion caused severe osteoporosis and reduced lifespan. These mice also showed elevated SASP expression in osteogenic and myeloid lineage cells that impaired osteogenesis and promoted osteoclastogenesis [31].

Interestingly, chronic accumulation of senescent cells would also appear to depend on exposure to ionizing radiation (IR), which increases bone resorption and reduces the biomechanical strength of bone. In a recent paper, SASP levels have been quantified in a mouse model exposed to IR, indicating a significant increase in the interleukin 1alpha (*IL-1α*), interleukin-1 beta (*IL-1β*), *IL-6*, *IL-8*, matrix metalloproteinase-3 (*MMP-3*), matrix metalloproteinase-13 (*MMP-13*), and *TNF-α*, in osteocytes of IR-irradiated mice compared to the control group. In addition, authors detected elevated expression of senescence markers such as p16, p21, and p53, concluding that IR exposure induces oxidative stress, causes DNA damage, and accelerates bone tissue senescence [32].

Noteworthy, osteoblast senescence also seems to be involved in the progression of osteoporosis. In this regard, it has been investigated whether activation of the vitamin D receptor (VDR) could regulate ferroptosis, reducing lipid peroxidation and counteracting age-related bone loss, in osteoblasts of VDR-knockout or D-galactose (D-gal)-induced mice. Not surprisingly, VDR activation inhibited ferroptosis through an up-regulation of erythroid nuclear factor 2-related factor 2 (Nrf2) and glutathione peroxidase 4 (GPX4), suggesting a key role for the VDR/Nrf2/GPX4 signaling pathway in counteracting osteoblastic senescence and age-related osteoporosis [33].

Finally, the accumulation of advanced glycation end-products (AGEs) would appear to play a key role in cellular senescence-induced changes and the development of age-related diseases [34]. In this regard, it has been observed that AGEs accelerate the senescence of bone marrow stromal cells (BMSCs) in a concentration-dependent manner by increasing the expression of senescence markers, including p16, p21, and p53 [35]. Furthermore, AGEs impair mitochondrial function by increasing levels of reactive oxygen species (ROS) and reducing mitochondrial membrane potential, as well as decreasing the expression of markers of mitophagy, including LC3B, Parkin, and sirtuin 3 (Sirt3). Interestingly, silencing Sirt3 worsened AGE-induced dysfunction, suggesting modulation of mitophagy as a potential strategy to prevent cellular aging [35].

**Figure 1:**
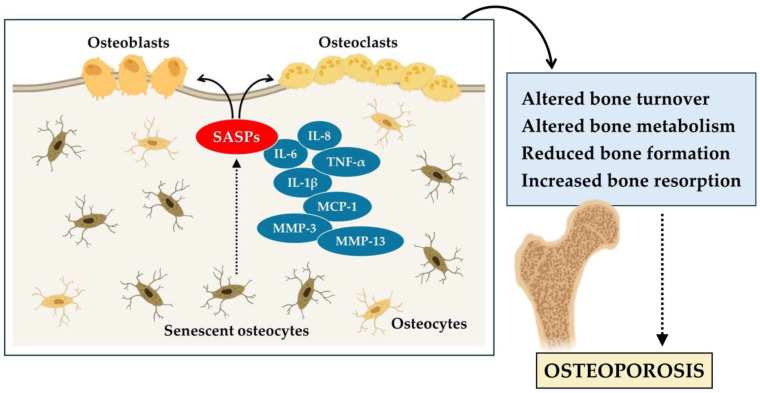
Cellular senescence contributes to the aging of bone tissue and the onset of osteoporosis. In osteoporosis, senescent osteocytes release various senescence-associated secretory phenotype (SASPs), such as interleukin-6 (IL-6), interleukin-8 (IL-8), interleukin-1 beta (IL-1β), tumor necrosis factor α (TNF-α), monocyte chemoattractant protein-1 (MCP-1), matrix metalloproteinase-3 (MMP-3), and matrix metalloproteinase-13 (MMP-13). These alter bone turnover, reducing bone formation by osteoblasts, increasing bone resorption by osteoclasts, and promoting the progression of osteoporosis.

In conclusion, these studies highlight the central role of senescent cells in age-related bone loss and osteoporosis. Senescent cells of the myeloid lineage and osteocytes are the main sources of SASP in the bone microenvironment, resulting in altered bone remodeling. Osteocyte senescence alters bone metabolism, while osteocyte depletion induces severe skeletal conditions such as osteoporosis. In addition, senescent osteoblasts contribute to the progression of osteoporosis (Figure 1). Therefore, the accumulation of senescent cells has a significant impact on bone health and accelerates skeletal aging.

### 2.2. Muscle Senescence in Sarcopenia

Sarcopenia is a multifactorial disorder caused by altered neuromuscular function, oxidative stress, and systemic inflammation, as well as mitochondrial dysfunction and hormonal imbalance [36]. The resulting decline in muscle mass and strength would appear to depend in part on the accumulation of senescent cells and SASPs in skeletal muscle, which alter the integrity and function of the local tissue by inducing paracrine senescence, stem cell dysfunction, and alterations in the extracellular matrix [37].

Among the proposed mechanisms, endothelial dysfunction associated with an increase in endothelin-1 (ET-1) synthesis could promote muscle senescence by altering the proliferative activity of stem cells. In this regard, it has been demonstrated that ET-1-induced senescence in the murine myoblast cell line induces fibronectin, which interacts with the integrin receptor by promoting the production of ROS and activating the phosphoinositide 3-kinase (PI3K)/tyrosine kinase B (AKT)/glycogen synthase kinase 3β (GSK) signaling pathway. In addition, in vivo measurements on 5-, 18- and 24-month-old male C57Bl6 mice showed high levels of circulating ET-1 and muscle fibrosis in aged mice compared to other experimental groups, in association with increased muscle expression of p16 and loss of muscle strength [38]. Similarly, Moustogiannis and colleagues analyzed the impact of myoblastic aging on skeletal muscle formation in vitro using a model simulating cellular senescence in C2C12 myoblasts [39]. Interestingly, a reduced expression of factors contributing to muscle growth, such as myogenic regulatory factors (MRFs), was observed in senescent myoblasts, as well as an over-expression of p53, forkhead box O1 (FoxO1) protein, and IL-6. Therefore, the reduction of myogenic potential and the induction of apoptotic, inflammatory, and atrophy factors could suggest a compromised myogenic lineage in senescent muscle cells [39].

Interestingly, 1,25(OH)_2_D deficiency has been proposed to promote cellular senescence and SASP release by Yu and colleagues, studying the musculoskeletal phenotype of wild-type (WT) and knockout mice for Cyp27b1 (KO), a key enzyme that promotes the synthesis of the active form of vitamin D [40]. Increased expression of protein and mRNA levels of p16, p21, TNF-α, IL-6, and MMP-3 was observed in the KO mice compared to the WT group, in association with muscle atrophy and decreased mitochondrial metabolic activity. Therefore, the authors suggested that 1,25(OH)_2_D deficiency may contribute to the development of age-related sarcopenia by causing oxidative stress, promoting skeletal muscle cell senescence, and hindering muscle regeneration [40].

Although the accumulation of senescent cells emerges as a determinant of the decline in muscle mass and strength associated with aging, muscle tissues, particularly myoblastic cells, are known to exhibit a distinctive panel of SASP markers. The senescent phenotype of myoblasts and fibroblasts isolated from muscle biopsies taken from healthy young adults was characterized after treatment with doxorubicin, a chemotherapeutic agent known to induce senescence [41]. Analysis of mRNA by qRT-PCR revealed distinct senescence phenotypes between the two cell types. Particularly, significantly elevated levels of *MMP-3* mRNA and *IL-8* were measured in myoblasts, while insulin-like growth factor-binding protein 3 (IGFBP3) showed increased expression in fibroblasts after prolonged incubation time with doxorubicin. Furthermore, some factors such as plasminogen-1 (PAI-1) and chemokine 5 (CXCL-5) were more highly expressed in fibroblasts, while TNF-α and IL-6 showed an opposite trend, suggesting senescence as a dynamic process characterized by complex inter- and intracellular variability [41].

Finally, it has been investigated the role of cellular senescence in muscle performance decline in a large cohort of elderly individuals, finding significant associations between SASP levels and measures of strength and mobility, such as the short physical performance battery (SPPB) and grip strength. Importantly, the strongest association was found between activin A, a negative regulator of muscle mass, and physical decline, confirming the key role of senescence markers in age-associated decline in physical performance [42].

**Figure 2:**
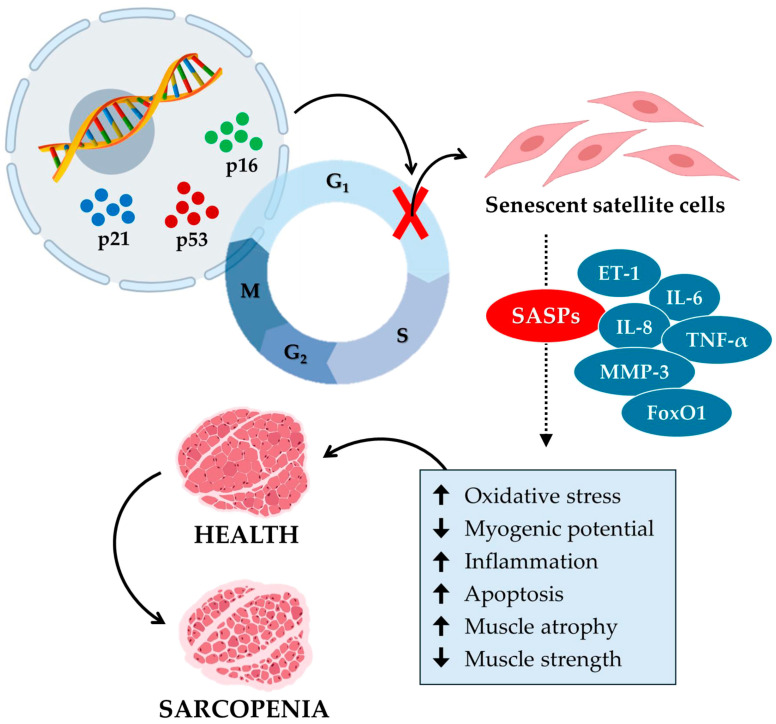
Cellular senescence contributes to the aging of muscle tissue and the onset of sarcopenia. Cellular senescence in sarcopenia is a process in which specific senescence markers, such as p16, p21, and p53, block the cell cycle by inhibiting the growth and proliferation of satellite cells. In turn, senescent satellite cells release various senescence-associated secretory phenotypes (SASPs), such as endothelin-1 (ET-1), interleukin-6 (IL-6), interleukin-8 (IL-8), tumor necrosis factor α (TNF-α), matrix metalloproteinase-3 (MMP-3), and forkhead box O1 (FoxO1). In muscle tissue, SASPs increase (up arrow) oxidative stress, inflammation, and apoptosis and reduce (down arrow) myogenic potential. Consequently, muscle atrophy increases, and muscle strength decreases, promoting the onset of sarcopenia.

Overall, these studies illustrate how senescence in satellite cells contributes to sarcopenia, emphasizing the role of mitochondrial function and SASP secretion in age-related loss of muscle mass and function (Figure 2). Therefore, the development of targeted interventions aimed at mitigating senescence and improving mitochondrial function appears to be necessary to combat sarcopenia and preserve muscle health in the aging population.

### 2.3. Cartilage Senescence in Osteoarthritis

A growing number of studies have documented age-associated changes in the extracellular matrix and cartilage cells, reporting reduced thickness, proteolysis, advanced glycation, and calcification among the main changes, in association with reduced cell density, cellular senescence, and altered anabolic responses. The combination of these changes exacerbates mechanical stress and tissue destruction, contributing to biomechanical dysfunction and the risk of osteoarthritis [23]. Not surprisingly, osteoarthritis is an age-related degenerative cartilage disease characterized by an increase of senescent chondrocytes in the cartilage. Chondrocytes are the only cell type present in articular cartilage and maintain the homeostasis of the extracellular matrix by synthesizing type II collagen, which confers considerable tensile strength [43]. Indeed, selective removal of senescent chondrocytes in a mouse model of post-traumatic osteoarthritis counteracted cartilage degeneration, while intra-articular injection of senescent cells induced osteoarthritis in mice [44].

Importantly, synovial macrophages, fibroblasts, and osteoclasts have been suggested to promote SASP production in the senescent microenvironment and trigger an inflammatory response that alters subchondral bone remodeling, leading to cartilage loss and disease progression [45,46]. Among these, monocyte chemoattractant protein 1 (MCP-1), interleukin 1 (IL-1), IL-6, MMP-3, and MMP-13 have been proposed as key mediators of the inflammation that characterizes osteoarthritis [47]. These factors contribute to an inflammatory microenvironment, which can be beneficial if the inflammation resolves quickly, as in cases of joint trauma. However, prolonged inflammation can be harmful, leading to chronic inflammation that causes senescence of neighboring healthy cells, as well as degradation of the extracellular matrix and synovitis. Finally, certain components of SASPs, such as TNF-α, IL-1, and IL-6, appear to be involved in the development of the pain typical of osteoarthritis, highlighting the need to investigate the underlying mechanisms [5].

Interestingly, the key role of senescent fibroblast-like synoviocytes (FLS) in the progression of osteoarthritis in vitro and in vivo has recently been emphasized, finding a positive correlation between FLS senescence and the presence of N6-methyladenosine (m^6^A), a post-transcriptional RNA modification known to be involved in DNA damage, autophagy, and cellular senescence [48,49]. Specifically, the authors observed that impaired autophagy in FLS accelerates cellular senescence through a GATA binding protein 4 (GATA4)-dependent mechanism, the levels of which were significantly elevated in osteoarthritic patients. Overall, autophagy prevents cellular senescence by suppressing GATA4, making it a potential therapeutic target for osteoarthritis [48].

Sirtuin 6 (Sirt6), a member of the sirtuin family of NAD+-dependent enzymes, also appears to play a central role in the pathogenesis of osteoarthritis by reducing the inflammatory response and chondrocyte senescence [50]. In this context, Ji and colleagues studied the effect of Sirt6 on chondrocyte senescence in vivo and in vitro, demonstrating a role in the regulation of the interleukin-15 (IL-15)/Janus kinase 3 (JAK3)/signal transducer and activator of transcription 5 (STAT5) signaling pathway [51]. Specifically, the authors observed that overexpression of Sirt6 significantly inhibits IL-15-induced STAT5 phosphorylation, attenuating IL-15/JAK3-induced translocation of STAT5 from the cytoplasm to the nucleus and subsequent chondrocyte senescence. Therefore, preferential activation of Sirt6 in chondrocytes could represent a novel and effective strategy to prevent and treat osteoarthritis [51]. More recently, it has been demonstrated that reduced levels of sirtuin 4 (Sirt4) impaired the cellular ability to eliminate damaged mitochondria by inhibiting PTEN-induced kinase 1 (Pink1) in chondrocytes, promoting oxidative stress and chondrocyte senescence. In contrast, upregulation of Sirt4 successfully counteracted mitochondrial dysfunction, improved mitophagy, and protected against chondrocyte senescence, representing a potential therapeutic target for the treatment of osteoarthritis [52].

Moreover, the infrapatellar fat pads and synoviocytes, which are also affected by senescence, become sources of inflammatory cytokines such as IL-1β and TNF-α. These cytokines not only promote local inflammation but also accelerate cartilage degeneration and subchondral bone remodeling [53]. In a study on rats, aging was associated with increased basal production of TNF-α and anti-inflammatory cytokines, such as interleukin-13 (IL-13), though pro-inflammatory cytokines were more prevalent. Aging also reduced the expression of M2 macrophage markers, indicating a heightened susceptibility to inflammation [54]. The interaction between synoviocytes and infrapatellar fat tissue thus triggers a vicious cycle of inflammation and joint degeneration, where pro-inflammatory cytokines activate metalloproteinases that degrade cartilage, and subchondral bone remodeling disrupts joint biomechanics. This vicious cycle drives the progression of osteoarthritis, exacerbating clinical symptoms such as pain and joint stiffness [55].

**Figure 3:**
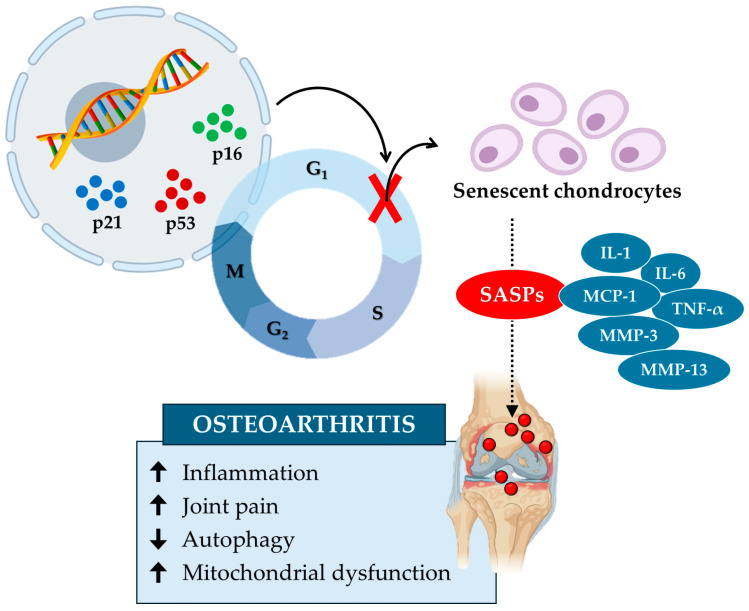
Cellular senescence contributes to cartilage tissue aging and the onset of osteoarthritis. Cellular senescence in osteoarthritis is induced by specific senescence markers, such as p16, p21, and p53, which block the cell cycle and inhibit chondrocyte growth and proliferation. In turn, senescent chondrocytes release various senescence-associated secretory phenotypes (SASPs), such as interleukin-1 (IL-1), interleukin-6 (IL-6), monocyte chemoattractant protein-1 (MCP-1), tumor necrosis factor α (TNF-α), matrix metalloproteinase-3 (MMP-3), and matrix metalloproteinase-13 (MMP-13). These factors increase (up arrow) inflammation, joint pain, and mitochondrial dysfunction while reducing (down arrow) autophagy by promoting the onset of osteoarthritis.

In conclusion, the accumulation of senescent chondrocytes alters the extracellular matrix and increases mechanical stress, leading to biomechanical dysfunction and an increased risk of osteoarthritis (Figure 3). Studies highlight the role of senescent microenvironments, characterized by synovial fibroblasts and macrophages producing SASPs, in exacerbating cartilage degeneration and inflammation. Key molecules such as GATA4 and sirtuins could represent potential therapeutic targets in the treatment of osteoarthritis, underlining the importance of thoroughly investigating the underlying biological mechanisms.

## 3. Anti-Senescence Strategies

**Figure 4:**
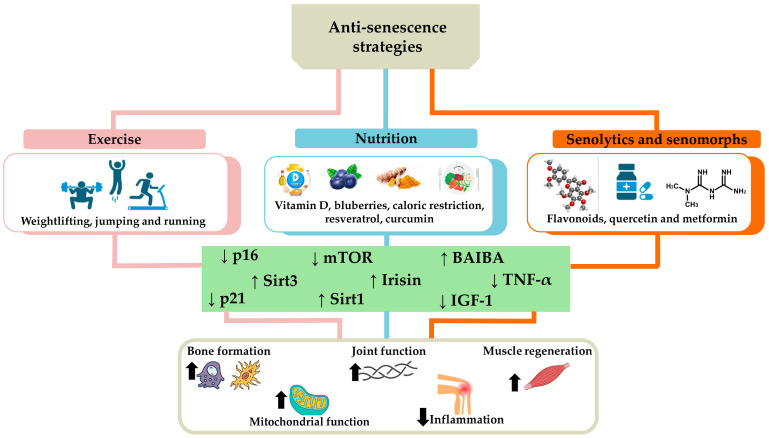
Anti-senescence strategies to counteract osteoporosis, sarcopenia, and osteoarthritis. The main strategies to counteract cellular senescence characterizing age-related musculoskeletal disorders include exercise, such as running, jumping, and weightlifting, and nutrition, rich in vitamin D, blueberries, resveratrol, curcumin, or based on calorie restriction. The use of senolytics and senomorphs, such as flavonoids, quercetin, and metformin, is also a useful tool to counteract senescent cell accumulation. Overall, these strategies promote tissue homeostasis by reducing (down arrow) the levels of p16, p21, tumor necrosis factor α (TNF-α), mammalian target of rapamycin (mTOR), and insulin-like growth factor-1 (IGF-1), as well as increasing (up arrow) the expression of sirtuin 1 (Sirt1), sirtuin 3 (Sirt3), irisin, and β-aminoisobutyric acid (BAIBA). Consequently, an increase in bone formation, joint function, muscle regeneration, and mitochondrial function has been observed in association with a reduction in inflammation, counteracting the onset and/or progression of osteoporosis, sarcopenia, and osteoarthritis.

Anti-senescence strategies combining exercise, nutrition, use of senolytics, and senomorphs offer an integrated approach to counteract osteoporosis, sarcopenia, and osteoarthritis. Particularly, regular exercise and a balanced diet provide a solid foundation for overall health, while senolytics and senomorphs represent innovative interventions aimed at removing or modifying senescent cells, reducing inflammation, and promoting tissue regeneration (Figure 4). These integrated approaches have the potential to significantly improve the quality of life of the elderly and slow the progression of degenerative diseases, although further research is needed to ensure their long-term safety and efficacy.

### 3.1. Exercise

Exercise, as an anti-senescence strategy, plays a crucial role in mitigating cellular senescence and preventing associated diseases. In fact, integrating regular physical activity into the daily routine is known to significantly improve bone, joint, and muscle health, delaying the onset of chronic diseases and improving the quality of life of the elderly [56].

Particularly, loading activities such as running, jumping, and weightlifting stimulate bone formation through mechanical stress, promoting the production of growth factors and the differentiation of mesenchymal stem cells (MSCs) in the osteoblastic line, resulting in an increase in osteocytes [57]. Although the underlying molecular mechanisms are largely unknown, irisin and β-aminoisobutyric acid (BAIBA), two exercise-induced myokines, would appear to directly slow down age-related osteocyte senescence by preserving mitochondrial function and the processes of biogenesis and mitophagy [58]. Sirt3, predominantly expressed in mitochondria, is also known to regulate energy metabolism, ROS homeostasis, and mitochondrial biogenesis [59]. Recent evidence has found a close association between the aging process and the reduction in the number of mitochondria within the dendritic processes of osteocytes, suggesting Sirt3 deficiency as the main culprit for inhibiting osteoblastic differentiation and reducing the positive effects of exercise on skeletal muscle [60,61]. In agreement, Li and colleagues demonstrated that inhibition of Sirt3 impairs the formation of osteocyte dendritic processes and inhibits bone formation in 20-month-old mice subjected to running exercises for six consecutive weeks. Interestingly, administration of honokiol, a Sirt3 activator, improved osteocyte sensitivity to mechanical stress in vitro, while intraperitoneal injection of honokiol reduced bone loss in aged mice in a dose-dependent manner, suggesting it as a potential therapeutic strategy to prevent osteoporosis and amplify the benefits of exercise on bone, especially in the elderly population [62].

Undoubtedly, exercise is the most effective tool for preventing the reduction in muscle mass and strength that characterizes sarcopenia, although the underlying processes have not been fully elucidated [63]. Furthermore, little evidence has investigated the mechanisms by which increased catabolism induced by aerobic exercise influences skeletal muscle atrophy and other senescence phenotypes, highlighting the existence of several gaps in this field [64]. In this respect, 5-aminoimidazole-4-carboxamide-1-β-D- ribofuranoside (AICAR), with similar action to that of exercise, was used to investigate the effects on energy metabolism in the C2C12 cell line [65]. Interestingly, AICAR-induced activation of adenosine monophosphate-activated protein kinase (AMPK) mitigated the reduced skeletal muscle cell viability caused by doxorubicin, a known inducer of senescence in vitro. Furthermore, four weeks of treadmill exercise attenuated the acceleration of skeletal muscle senescence in aged mouse models by reducing the expression levels of p16 and p21. Therefore, exercise or an environment mimicking AICAR-induced exercise could delay skeletal muscle cell senescence typical of sarcopenia [65]. Similarly, Englund et al. investigated the effects of progressive strength and endurance training 2 days per week for 12 weeks on the molecular phenotype of CD3+ T-cells and circulating concentrations of SASP in a group of elderly people, observing significant reductions in the expression of p16, p21, TNF-α and other mediators of inflammation. Importantly, the trained elderly showed a significant improvement in muscular performance and physical function in terms of grip strength, repetitive sit-up-and-go time, and timed up-and-go (TUG) testing, suggesting the structured exercise program as a valid strategy to counteract age-related cellular senescence [66].

Finally, exercise is the first line of non-surgical treatment for osteoarthritis, improving pain and function, reducing physical impairment, and increasing the quality of life [67,68]. However, although low- to moderate-intensity exercise is useful in counteracting cartilage degeneration and chondrocyte apoptosis, the role of exercise in preventing the progression of osteoarthritis with aging is still highly debated, and its effects have not been fully investigated [69]. In this regard, it was recently investigated whether exercise could alleviate age-associated symptoms of knee osteoarthritis in a senescent male accelerated prone 8 (SAMP8) mouse model, characterized by a mutated mitochondrial genome causing increased oxidative stress and premature aging [70]. Importantly, treadmill running at a speed of 10 m/min for 15 min/day for 3 days did not attenuate cartilage degeneration in the medial and posterior tibial plateau. However, an increase in type II collagen levels in the remaining cartilage was found, in association with suppression of synovial inflammation and reduced infiltration of pro-inflammatory macrophages [70].

In vitro studies have shown that human chondrocytes or cartilage explants subjected to mechanical stimuli show different regulation of matrix proteins, cytokines, growth factors, and degradation enzymes, with often conflicting results [71]. In this context, Jørgensen et al. conducted a randomized controlled trial to investigate the effects of a single endurance exercise session on gene expression and glycosaminoglycan (GAG) content in the cartilage of patients with primary osteoarthritis of the knee, finding elevated expression of *MMP-3* and *MMP-13* in the submeniscal area [72]. Noteworthy, the expression of matrix metalloproteinase-1 (*MMP-1*), *MMP-13*, and *TGF-β* was higher in osteophytes than in articular cartilage, while a minimal effect was found on GAG composition. Thus, the differences in expression in the regions examined suggest a chronic mechanical influence on human cartilage [72].

Overall, regular exercise has significant beneficial effects in counteracting age-related senescence. In fact, a crucial role for exercise has been widely demonstrated in preventing osteoporosis by increasing bone density and reducing the risk of fractures, improving joint function and reducing the symptoms of osteoarthritis, as well as counteracting sarcopenia by preserving muscle mass and strength, improving balance and reducing the risk of falls in the elderly. However, further studies are needed to explore in detail the biological mechanisms underlying these effects, which remain partly unknown.

### 3.2. Nutrition

Nutrition plays a crucial role in the prevention of age-related musculoskeletal disorders, as the intake of essential nutrients such as calcium, vitamin D, protein, and antioxidants is crucial for maintaining bone and muscle health [73]. In fact, a balanced diet helps preserve bone density, preventing osteoporosis, and maintain muscle mass and strength, reducing the risk of sarcopenia. In addition, specific nutrients can have anti-inflammatory effects that help prevent and manage osteoarthritis, improving mobility and overall quality of life [74]. For this reason, current research has focused on identifying a correlation between nutrition and cellular senescence in an attempt to identify a type of diet that can modulate the biological processes underlying cellular senescence, contributing to healthier aging and slowing the onset of age-related diseases [75].

Calorie restriction without malnutrition is currently the most effective treatment in delaying aging and reducing cellular senescence, being known to reduce calorie intake by 30–40% while maintaining the right balance of macronutrients [76]. In this context, the effect of calorie restriction on the quality of life in healthy subjects randomized to 2 years of 25% calorie restriction has been tested, and an improvement in mood, quality of life, sleep, and sexual function was observed compared to the control group [77]. Noteworthy, certain natural compounds, including oleuropein, curcumin, and essential minerals such as magnesium and selenium, are known to mimic the effects of calorie restriction by stimulating longevity pathways [78,79,80,81]. Among these, activation of AMPK and sirtuins, as well as inhibition of insulin-like growth factor-1 (IGF-1) and mammalian target of rapamycin (mTOR), would appear to be the main target pathways involved, although the underlying molecular mechanisms that reduce cellular senescence need further investigation [82]. Particularly, resveratrol is known to enhance sirtuin 1 (Sirt1)-mediated deacetylation, promoting osteoblast differentiation and inhibiting osteoclast formation [83]. Wang and colleagues found that high-dose resveratrol administration was correlated with reduced bone loss in ovariectomized rats compared to untreated controls, in association with elevated serum levels of osteoprotegerin and β-catenin and reduced expression of nuclear factor kappa-light-chain-enhancer of activated B cells (NF-κB). Therefore, incorporating resveratrol into the diet may support Sirt1 activity and help mitigate bone senescence [84]. Importantly, curcumin, especially its analog UBS109, has demonstrated considerable potential in treating osteoporosis by promoting osteoblast differentiation, enhancing mineralization, and inhibiting osteoclastogenesis. UBS109 also influences Smad signaling and suppresses NF-κB activation, thereby counteracting bone resorption [85]. In addition, natural supplements such as orthosilicic acid and vitamin K2 have been shown to enhance bone health by stimulating osteoblast activity and reducing inflammation. Together, these natural compounds not only support osteogenesis but also combat inflammation associated with cellular senescence, making them particularly valuable in the early prevention and treatment of osteoporosis. By upregulating key osteogenic markers like alkaline phosphatase (ALP) and collagen type I (Col1α1), these compounds represent a promising strategy for managing osteoporosis [86].

A diet low in vitamin D would also appear to contribute to premature aging of the body, given its involvement in the maintenance of genomic stability, cell cycle arrest, and modulation of SASPs [87]. Indeed, a diet rich in vitamin D is known to benefit sarcopenia and frailty by regulating the mTOR signaling pathway, which is impaired in senescent cells [88].

Interestingly, blueberries have been suggested to promote osteoblastic differentiation due to their folic acid content, and their consumption in the early years of life appears to delay the aging process and prevent bone loss in adults [89,90]. In this regard, a blueberry diet has been shown to prevent collagen loss in the bone matrix and inhibit senescence in osteoblastic cells of ovariectomized rats by increasing the expression of Sirt1 and reducing the levels of senescence markers such as p16, p21, and p53 [91].

Finally, a close association has been proposed between changes in the gut microbiota and the accumulation of senescent cells, leading to a decline in biological functions and the development of age-related diseases [92]. Indeed, the gut microbiota, crucial for immunity and metabolism, becomes dysbiosis with age, affecting overall health [93]. Furthermore, commensal bacteria induce intestinal B-cell senescence, reducing IgA production and causing dysbiosis, as well as negatively influencing the survival and maintenance of musculoskeletal cells and impairing immune function [94]. Therefore, monitoring microbial metabolites could help in the diagnosis of dysbiosis and the prevention of age-related musculoskeletal disorders. Furthermore, understanding the underlying mechanisms could promote the development of strategies to prevent the induction of cellular senescence through modulation of the gut microbiota. However, further studies are necessary to clarify these interconnections and develop targeted interventions for healthy aging [95].

### 3.3. Senolytics and Senomorphs

Cellular senescence has been identified as a particularly promising therapeutic target to mitigate the effects of aging and improve overall health [96]. Current scientific literature focuses on treatment with senolytics and senomorphs to remove senescent cells from the musculoskeletal system, thus delaying the aging process in age-related diseases [97]. Specifically, senolytics are a class of drugs designed to selectively remove senescent cells, while senomorphs are compounds that inhibit the secretion of SASPs by attenuating the harmful effects of senescent cells on the environment, reducing inflammation, and slowing the progression of age-related diseases. Together, senolytics and senomorphs represent two complementary therapeutic strategies to address cellular senescence and promote healthier aging [98].

Senolytics such as dasatinib and quercetin have proven effective in counteracting cellular aging by selectively targeting senescent cells and attenuating SASPs in multiple murine tissues [99,100]. Estrogen-deficiency-induced bone loss in middle-aged mice has recently been observed to depend largely on inflammatory processes with SASPs release and accelerated accumulation of senescent cells, especially senescent MSCs [101]. Surprisingly, systemic administration of a senolytic drug containing dasatinib and quercetin (DQ) reduced the accumulation of senescent cells, restoring MSC function and promoting bone formation. Furthermore, local administration of DQ combined with bone morphogenetic protein 2 (BMP-2), a known osteoinductive agent, showed synergistic effects on bone regeneration, confirming the efficacy of DQ in the treatment of post-menopausal osteoporosis [101]. Importantly, the study conducted by Liu et al. extended the use of senolytics to age-related fractures, showing that short-term administration of DQ promoted fracture healing in 20-month-old mice. Furthermore, the injection of TGF-β neutralizing antibodies improved fracture healing by increasing the number of CaMPCs, confirming senolytic drugs as a promising therapy in counteracting age-related cellular senescence [28].

Among natural flavonoids, fisetin has demonstrated potent senotherapeutic effects, reducing senescence markers and inflammation in various tissues [102]. In this context, it has been demonstrated that acute and chronic treatment with fisetin in progeroid and WT mice reduced senescence markers in various tissues by specifically attacking cells expressing p16. Furthermore, fisetin reduced the fraction of senescent T lymphocytes and natural killer cells, as well as markers of inflammation and oxidative stress, highlighting its potential to restore tissue homeostasis and promote general health [103].

In the field of osteoarthritis, several senomorphs have been tested to counteract cellular senescence. Among them, lutikizumab, canakinumab, tocilizumab, etanercept, and CL82198 are known to directly inhibit or neutralize SASPs, including IL-1β, IL-6, TNF-α, and MMP-13 [104,105]. Furthermore, quercetin has been suggested to counteract cartilage degradation and apoptosis of chondrocytes in 8-week-old rats by reducing oxidative stress, restoring mitochondrial membrane potential, and inhibiting the caspase-3 pathway [106]. Metformin has also emerged as a promising candidate to regulate senescence and treat osteoarthritis, as it appears to act through multiple mechanisms, including activation of AMPK and Sirt1, downregulation of mammalian target of rapamycin complex 1 (mTORC1), inhibition of NF-κB, and enhancement of metabolism [107].

Overall, senolytics and senomorphs acting on cellular senescence could offer a comprehensive approach to treating age-related diseases and promoting healthier aging, suggesting the need for further studies to fully understand and validate their use in clinical settings.

## 4. Conclusions

The review highlights several key limitations, including variability in experimental models and the challenge of translating findings from animal studies to humans, given that the number of clinical studies in humans is currently limited. Additionally, the reliability of senescence biomarkers may be affected by non-specific factors. Nevertheless, cellular senescence has emerged as a key factor in the pathophysiology of age-related musculoskeletal diseases, including osteoporosis, sarcopenia, and osteoarthritis. Recent research highlights the profound impact of senescent cell accumulation on bone, muscle, and cartilage health, accelerating skeletal aging and aggravating degenerative conditions. Indeed, senescent cells release SASPs that promote bone resorption, disrupt muscle regeneration, and contribute to joint degeneration. Undoubtedly, therapeutic strategies aimed to remove senescent cells or modulate their signaling pathways represent a promising approach for the management of these diseases. However, an understanding of the underlying mechanisms remains incomplete, complicating the development of precise and targeted interventions. Current therapeutic approaches include regular exercise, a balanced diet, and the use of senolytics and senomorphs, which attenuate cellular senescence and potentially slow the progression of musculoskeletal diseases. Noteworthy, cellular senescence remains a physiological process that accompanies aging. Therefore, interventions such as regular exercise and proper nutrition not only have the potential to manage these diseases but also to prevent or slow the onset of cellular senescence itself. Future research focused on elucidating the mechanisms of aging will be crucial to refine these therapeutic strategies and improve outcomes for patients with age-related musculoskeletal diseases.

## Data Availability

No new data were created or analyzed in this study. Data sharing is not applicable to this article.
